# Emotional and intuitive eating: an emerging approach to eating behaviours related to obesity

**DOI:** 10.1017/jns.2023.11

**Published:** 2023-02-13

**Authors:** Feride Ayyıldız, Gamze Akbulut, Merve Şeyda Karaçil Ermumcu, Nilüfer Acar Tek

**Affiliations:** 1 Department of Nutrition and Dietetics, Gazi University Faculty of Health Sciences, Ankara, Turkey; 2 Department of Nutrition and Dietetics, Akdeniz University Faculty of Health Sciences, Antalya, Turkey

**Keywords:** Anthropometric measurement, Emotional eating, Intuitive eating, Obesity, Obesity-related diseases

## Abstract

Emotional and intuitive eating are associated with obesity. In the present study, it was aimed to evaluate the relationship between intuitive eating and emotional eating behaviours in adults with anthropometric measurements of obesity-related disease risk and gender. Body weight, body mass index (BMI), waist, hip and neck circumferences were taken. Emotional Eater Questionnaire and Intuitive Eating Scale-2 were used for the assessment of eating behaviour. A total of 3742 adult individuals (56⋅8 % (*n* 2125) female and (*n* 1617) male) were participated voluntarily. The total score and subscales of EEQ were higher in females than males (*P* < 0⋅001). The scores of the IES-2 subscales and the total score were higher in males than females (*P* < 0⋅05). In metabolic risk classification according to waist and neck circumference, EEQ scale scores (except type of food) were higher in the metabolic risk group, while IES-2 (except body-food congruence in neck circumference) scores were higher in the non-risk group (*P* < 0⋅05). While there was a positive correlation between EEQ and body weight, BMI, waist circumference, waist-height ratio, a negative correlation was found between age and waist-hip ratio. There was a negative correlation between IES-2 and body weight, BMI, waist-height ratio, waist-hip ratio. In addition, a negative correlation was found between IES-2 and EEQ. Intuitive eating and emotional eating differ by gender. Anthropometric measures and metabolic disease risk is associated with emotional eating and intuitive eating. Interventions to increase intuitive and decreasing emotional eating behaviour can be effective in preventing both obesity and obesity-related diseases.

## Introduction

Obesity, which is an increasing prevalence worldwide, has many reasons such as age, gender, insufficient physical activity, dietary intake and eating behaviour. Nowadays in particular, the eating behaviours of individuals also play an active role in the development and prevention of obesity and obesity-related disease^(1)^. Obesity is associated with the risk of many diseases (cardiovascular diseases, diabetes mellitus, etc.) and anthropometric measurements being within the ideal limits are associated with a decrease in the risk of disease^(2–4)^. Especially changes in eating behaviour can cause obesity and are also important in preventing obesity^(5)^. So it may be necessary to assess the eating behaviour both to prevent the development of obesity and to reduce obesity-related diseases.

Eating, which is a biological need, is not just a state of hunger. Eating behaviour is affected by many factors such as genes, hormones, religious beliefs, mood, media and environment and it was shown that there is an important relationship between eating behaviour and food intake^(6)^. In cases where there is no physiological hunger, individuals may want to eat when they see palatable foods^(7,8)^. The tendency to consume calorie-dense and high-fat foods, which are among the important dietary factors in the development of obesity, increases in people with emotional problems^(9)^. In recent years, some eating behaviours have been emphasised in order to be aware of and cope with the changes in food consumption depending on the emotional states of individuals. Intuitive and emotional eating are eating behaviours that are emphasised in the prevention of obesity^(10)^. Some people may be more sensitive to external stimuli such as the taste and visibility of food, regardless of hunger and satiety. This can lead to gain in body weight due to overeating^(11)^. While some eating behaviours can increase the response to both internal and external stimuli, some eating behaviours can also decrease it^(8)^.

Emotional eating (EE) was associated with weight gain is defined as the tendency to overeat with the effect of negative emotions^(12)^. Emotional eating can be affected by gender, socio-cultural and environmental factors as well as emotional state^(12)^. When evaluated according to gender, it has been reported that EE is more common in females than males^(13)^. Emotional eaters have been shown to be less successful in weight loss programmes than non-emotional eaters^(14)^. It is thought that a detailed examination of the factors affecting emotional eating may be effective in preventing obesity^(12)^.

Intuitive eating (IE) decreases energy intake by helping decrease in the response to both internal and external stimuli^(8)^. IE is defined as the individual's listening to physical hunger and satiety signals and adapting to these signals, reducing the sensitivity to emotions and thoughts during food intake^(15)^. IE has been shown to be negatively related to obesity and positively related to body image^(8)^. Also, there is no certain data about the difference of intuitive eating according to gender. A study was reported that females tend to lower levels of intuitive eating than males^(10)^. Other reported that IE does not differ by gender^(16)^.

The gender differences between EE and IE, which are important roles in weight control, are not clear. In addition, no study has been found on the evaluation of IE and EE, and the risk of obesity related to anthropometric measurements to our knowledge. In the present study, it was aimed to evaluate the relationship between IE and EE behaviours in adults with anthropometric measurements of obesity-related disease risk and gender.

## Materials and methods

### Subject

This cross-sectional study was conducted in Ankara, Turkey, between January and October 2019. The researchers reached adult individuals who live in the central districts of Ankara by the random sampling method. Clear explanations were provided to participating individuals regarding the purpose of the study, and those who wanted to participate in the study were included. Individuals with psychological disorders, using psychotherapeutic drugs, pregnant, following a weight loss programme and non-voluntary individuals were excluded from the study. According to the results of the power analysis conducted to determine this study's sample, it aimed to reach at least 1084 male and female individuals in the 95 % confidence interval. The target sample was doubled and a total of 3742 adult individuals [females: 56⋅8 % (*n* 2125) and males: (*n* 1617)] voluntarily were participated in the study. A total of 3742 adults [56⋅8 % (*n* 2125) of female and 43⋅2 % of male (*n* 1617), participated in the study. While the mean age of male and female were 40⋅9 ± 13⋅62 and 37⋅5 ± 13⋅90 years, respectively.

### Measures

#### Anthropometric measurements

Anthropometric measurements were applied to the participants with a face-to-face interview technique. The body weight (kg) was measured with an electronic scale and height was measured by a stadiometer. Body Mass Index (BMI) was calculated using the body weight/height (kg/m^2^) equation and classified as four groups as ‘underweight (<18⋅5 kg/m^2^), normal (18⋅5–24⋅99 kg/m^2^), overweight (25⋅0–29⋅9 kg/m^2^) and obese (≥30 kg/m^2^)’ according to the World Health Organization (WHO)^(17)^.

Similarly, the measurement method for waist circumference and hip circumference was taken from the report of the WHO. The report shows that if the waist circumference and waist-hip ratio increases, the risk of chronic disease also increases (waist circumferences’ cut-off: for female (F): 80 cm, male (M): 94 cm; waist-hip ratios’ cut-off F: 0⋅85, M: 0⋅90)^(3)^. In addition, waist-height ratio, which is a health risk indicator in adults, was calculated. Ashwell *et al.*^(2)^ stated that if this value is ≥0⋅5, early health risk increases. The neck circumference is associated with cardio-metabolic risk factors. We used neck circumference cut-offs of ≥37 cm in males and ≥33 cm in females in the evaluation of this risk factor^(4)^.

#### Emotional Eater Questionnaire (EEQ)

Emotional Eater Questionnaire (EEQ) was developed by Garaulet *et al.*^(18)^ to evaluate the emotional eating behaviours of individuals. Turkish validity and reliability of the scale was made by Akın *et al.*^(19)^. The Cronbach's alpha value of the scale is 0⋅88. In this study, it was found as 0⋅72. The scale consists of 10 items and 3 subscales (disinhibition (4, 5, 6, 8, 9 and 10 items), type of food (2 and 3 items) and guilt (1 and 7 items). The scale is evaluated according to a 4-point Likert type scale [from 0 (never) to 3 (always)]. High scores on the scale indicate a high level of emotional eating behaviour. Total score classified four groups as 0–5: non-emotional eater, 6–10: low emotional eater, 11–20: emotional eater, 21–30: very emotional eater^(18)^.

#### Intuitive Eating Scale-2 (IES-2)

Tylka *et al.*^(20)^ developed Intuitive Eating Scale-2 for evaluation of intuitive eating behaviour. Turkish validity and reliability of the scale was made by Bas *et al.*^(21)^. The Cronbach's alpha of Turkish version was 0⋅82 and 0⋅85 in the present study. This scale includes twenty-three items and four subscales: (a) eating for physical rather than emotional reasons, (b) unconditional permission to eat, (c) reliance on hunger and satiety cues and (d) body-food choice congruence. The scale is scored according to a 5-point Likert scale (1 = strongly disagree, 5 = strongly agree). Higher scores indicate a higher tendency to more intuitive eating.

### Statistical analysis

The data were evaluated in SPSS 22⋅0 statistical package program. The information about the categorical variables of the individuals is given in terms of frequency and percentage, and differences were examined with chi-square (*χ*^2^) analysis. For the assessment of quantitative data, mean 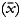 and median, standard deviation (sd) was calculated. Two group differences and more than two group differences were assessed using independent samples *t* test and one-way analysis of variance (ANOVA) test. The correlation between scale scores and anthropometric measurements was used Pearson correlation coefficient. All examinations were made statistically and interpreted at a 95 % confidence level (*P* < 0⋅05).

## Results

More than half of male (75⋅6 %) and female (76⋅5 %) were high school graduates and above. Besides, 62⋅2 % of male and 28⋅8 % of female work in a workplace. The 4⋅2 % of the participants were underweight, 47⋅5 % were normal weight, 33⋅5 % were overweight and 14⋅8 % were obese according to the BMI classification. In addition, the 59⋅5 % of males and 39⋅7 % of females were pre-obese or obese according to the BMI classification.

The evaluation of anthropometric measurements with EEQ and IES-2 by gender is given in Table 1. The total score and subscales of EEQ are higher in females than males (*P* < 0⋅001). The scores of the IES-2 subscales and the total score are statistically higher in males than females (*P* < 0⋅05). All evaluated anthropometric measurements (body weight, height, waist circumference, hip circumference and neck circumference) and calculated ratios (BMI, waist-hip ratio and waist-height ratio) were higher in male individuals (*P* < 0⋅05).

**Table 1.** Evaluation of some anthropometric measurements with EEQ and IES-2 by gender

	Male (*n* 1617)	Female (*n* 2125)	
	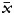 ± sd	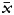 ± sd	*P*
EEQ			
Disinhibition	4⋅4 ± 3⋅68	5⋅2 ± 3⋅95	<0⋅001
Guilt	1⋅2 ± 1⋅37	2⋅1 ± 1⋅57	<0⋅001
Type of food	2⋅1 ± 1⋅40	2⋅7 ± 1⋅55	<0⋅001
Total score	7⋅8 ± 5⋅25	10⋅0 ± 5⋅80	<0⋅001
IES-2			
Eating for physical rather than emotional reasons	29⋅1 ± 6⋅21	27⋅5 ± 6⋅56	<0⋅001
Unconditional permission to eat	20⋅3 ± 4⋅15	19⋅3 ± 4⋅20	<0⋅001
Reliance on hunger and satiety cues	21⋅7 ± 5⋅24	21⋅4 ± 5⋅14	0⋅034
Body-food choice congruence	10⋅1 ± 2⋅61	10⋅0 ± 2⋅37	0⋅025
Total score	81⋅4 ± 12⋅12	78⋅2 ± 11⋅48	<0⋅001
Anthropometric measurements			
Body weight (kg)	80⋅1 ± 13⋅49	65⋅6 ± 13⋅31	<0⋅001
Height (cm)	174⋅5 ± 7⋅40	163⋅2 ± 6⋅42	<0⋅001
Waist circumference (cm)	92⋅1 ± 13⋅07	81⋅7 ± 14⋅18	<0⋅001
Hip circumference (cm)	100⋅5 ± 10⋅1	99⋅6 ± 11⋅44	0⋅010
Neck circumference (cm)	36⋅9 ± 3⋅46	33⋅2 ± 3⋅08	<0⋅001
BMI (kg/m^2^)	26⋅1 ± 4⋅09	24⋅6 ± 5⋅09	<0⋅001
Waist-hip ratio	0⋅9 ± 0⋅09	0⋅8 ± 0⋅08	<0⋅001
Waist-height ratio	0⋅5 ± 0⋅07	0⋅5 ± 0⋅09	<0⋅001

Independent samples *t* test.

EEQ, Emotional Eater Questionnaire; IES-2, Intuitive Eating Scale-2; BMI, body mass index.

The evaluation of EEQ and IES-2 according to the BMI classification is given in Table 2. Total score and the scores of other subgroups except type of the food in EEQ are higher in obese individuals than in other BMI classes (*P* < 0⋅001). The score of eating for physical rather than emotional reasons was lower in obese individuals (*P* < 0⋅001). In underweight individuals, the score of unconditional permission to eat was higher than other BMI classes (*P* < 0⋅001). The score of reliance on hunger and satiety cues were significantly both underweight and normal weight individuals than other groups (*P* < 0⋅001). Total score of IES-2 was similar in underweight and normal weight individuals and higher than overweight and obese individuals.

**Table 2.** The evaluation of EEQ and IES-2 according to the BMI classification

	BMI Classification				
	Underweight (*n* 159)	Normal weight (*n* 1777)	Overweight (*n* 1253)	Obese (*n* 553)	*P*
EEQ					
Disinhibition	4⋅0 ± 3⋅79^a^	4⋅4 ± 3⋅64^a^	4⋅9 ± 3⋅81^b^	6⋅3 ± 4⋅23^c^	<0⋅001
Guilt	1⋅0 ± 1⋅32^a^	1⋅6 ± 1⋅52^b,c^	1⋅7 ± 1⋅54^c^	2⋅1 ± 1⋅56^d^	<0⋅001
Type of food	2⋅8 ± 1⋅53^a^	2⋅4 ± 1⋅52^b,c^	2⋅3 ± 1⋅50^b,c^	2⋅5 ± 1⋅53^c^	<0⋅001
Total score	7⋅8 ± 5⋅27^a^	8⋅5 ± 5⋅46^a^	9⋅1 ± 5⋅64^b^	11⋅0 ± 6⋅10^c^	<0⋅001
IES-2					
Eating for physical rather than emotional reasons	29⋅4 ± 6⋅61^a^	28⋅4 ± 6⋅55^a^	28⋅2 ± 6⋅24^a^	27⋅0 ± 6⋅48^b^	<0⋅001
Unconditional permission to eat	21⋅7 ± 4⋅56^a^	19⋅7 ± 4⋅42^b^	19⋅4 ± 3⋅91^b^	19⋅8 ± 3⋅93^b^	<0⋅001
Reliance on hunger and satiety cues	22⋅9 ± 5⋅33^a^	22⋅0 ± 4⋅92^a^	21⋅1 ± 5⋅39^b^	20⋅6 ± 5⋅28^b^	<0⋅001
Body-food choice congruence	10⋅3 ± 2⋅68	10⋅1 ± 2⋅47	9⋅9 ± 2⋅48	10⋅0 ± 2⋅45	0⋅080
Total score	84⋅4 ± 11⋅75^a^	80⋅4 ± 11⋅91^b^	78⋅9 ± 11⋅91^c^	77⋅5 ± 11⋅09^c^	<0⋅001

ANOVA test.

*Same letters mean *P* > 0⋅05, different letters mean *P* < 0⋅05.

The evaluation of anthropometric measures of the individuals with regard to emotional eating classification is shown in Table 3. Anthropometric measurements were higher in male emotional eaters than in non-emotional eaters. There was no difference in waist-hip ratio between the groups in male individuals (*P* > 0⋅05). Body weight, BMI, waist circumference and waist-height ratio in female individuals were higher in emotional eaters than non-emotional eaters (*P* < 0⋅001). There was no difference between emotional eaters and non-emotional eaters in both waist-hip ratio and neck circumference in female individuals (*P* > 0⋅05).

**Table 3.** Evaluation of anthropometric measures of individuals with regard to the emotional eating classification

	Male (*n* 1617)					Female (*n* 2125)				
	1 (*n* 623)	2 (*n* 532)	3 (*n* 432)	4 (*n* 30)	*P*	1 (*n* 519)	2 (*n* 658)	3 (*n* 852)	4 (*n* 96)	*P*
Body weight (kg)	77⋅5 ± 12⋅23^a^	80⋅4 ± 12⋅89^b^	82⋅5 ± 15⋅14^b^	92⋅2 ± 10⋅00^c^	<0⋅001	62⋅2 ± 12⋅78^a^	64⋅4 ± 12⋅77^b^	67⋅8 ± 13⋅15^c^	71⋅9 ± 15⋅36^d^	<0⋅001
BMI (kg/m^2^)	25⋅4 ± 3⋅61^a^	26⋅1 ± 3⋅85^b^	27⋅0 ± 4⋅71^c^	30⋅2 ± 3⋅78^d^	<0⋅001	23⋅6 ± 4⋅96^a^	24⋅2 ± 5⋅06^a^	25⋅3 ± 5⋅00^b,c^	26⋅4 ± 5⋅47^c^	<0⋅001
Waist circumference (cm)	90⋅8 ± 12⋅63^a^	92⋅3 ± 12⋅64^a,b^	93⋅2 ± 14⋅08^b,c^	99⋅2 ± 10⋅37^c^	<0⋅001	79⋅7 ± 13⋅5^a^	80⋅5 ± 13⋅75^a^	83⋅4 ± 14⋅38^b,c^	85⋅7 ± 16⋅19^c^	<0⋅001
Neck circumference (cm)	36⋅7 ± 3⋅32^a^	36⋅9 ± 3⋅39^a^	37⋅0 ± 3⋅75^a^	38⋅7 ± 2⋅61^b^	0⋅008	32⋅8 ± 2⋅88^a^	33⋅0 ± 2⋅83^a,b^	33⋅4 ± 3⋅37^b^	33⋅6 ± 2⋅85^a,b^	<0⋅001
Waist-hip ratio	0⋅9 ± 0⋅09	0⋅91 ± 0⋅09	0⋅91 ± 0⋅09	0⋅9 ± 0⋅07	0⋅641	0⋅8 ± 0⋅08^a,b^	0⋅8 ± 0⋅80^a^	0⋅8 ± 0⋅09^b^	0⋅8 ± 0⋅08^a,b^	0⋅018
Waist-height ratio	0⋅5 ± 0⋅07^a^	0⋅5 ± 0⋅07^a,b^	0⋅5 ± 0⋅08^b,c^	0⋅5 ± 0⋅07^c^	<0⋅001	0⋅4 ± 0⋅08^a^	0⋅4 ± 0⋅09^a^	0⋅5 ± 0⋅10^b^	0⋅5 ± 0⋅09^b^	<0⋅001

ANOVA test 1: Non-emotional eater, 2: Low emotional eater, 3: Motional eater, 4: Very emotional eater.

EEQ, Emotional Eater Questionnaire; IES-2, Intuitive Eating Scale-2; BMI, body mass index.

*Same letters mean *P* > 0⋅05, different letters mean *P* < 0⋅05.

Evaluation of EEQ and IES-2 according to metabolic risk classification is given in Table 4. In metabolic risk classification according to waist and neck circumference, EEQ scale scores (except type of food) were higher in the metabolic risk group, while IES-2 (except body-food congruence in neck circumference) scores were higher in the non-risk group (*P* < 0⋅05). While EEQ scale scores (guilt and type of food) were higher in the non-metabolic risk group in risk in accordance with waist-hip ratio, but there was no significant difference between the groups in IES-2 scale scores. EEQ scale score, except type of food, was higher in the group with metabolic risk according to waist-height ratio, IES-2 scores (except eating for physical rather than emotional reasons) were higher in the non-risk group.

**Table 4.** Evaluation of EEQ and IES-2 according to the metabolic risk classification

	Waist circumference (cm)			Neck circumference (cm)			Waist-hip ratio			Waist-height ratio		
	No Metabolic Risk (*n* 1967)	Metabolic Risk (*n* 1775)	*P*	No Metabolic Risk (*n* 1760)	Metabolic Risk (*n* 1982)	*P*	No Metabolic Risk (*n* 2070)	Metabolic Risk (*n* 1672)	*P*	No Metabolic Risk (*n* 1737)	Metabolic Risk (*n* 2005)	*P*
EEQ												
Disinhibition	4⋅4 ± 3⋅65	5⋅3 ± 4⋅02	<0⋅001	4⋅5 ± 3⋅66	5⋅1 ± 4⋅00	<0⋅001	4⋅8 ± 3⋅82	4⋅9 ± 3⋅89	0⋅607	4⋅5 ± 3⋅71	5⋅1 ± 3⋅96	<0⋅001
Guilt	1⋅5 ± 1⋅48	1⋅9 ± 1⋅57	<0⋅001	1⋅6 ± 1⋅50	1–8 ± 1⋅56	<0⋅001	1⋅8 ± 1⋅55	1⋅6 ± 1⋅52	0⋅036	1⋅6 ± 1⋅51	1⋅8 ± 1⋅56	<0⋅001
Type of food	2⋅4 ± 1⋅52	2⋅5 ± 1⋅52	0⋅114	2⋅4 ± 1⋅51	2⋅4 ± 1⋅53	0⋅789	2⋅5 ± 1⋅52	2⋅3 ± 1⋅51	<0⋅001	2⋅5 ± 1⋅53	2⋅3 ± 1⋅51	0⋅001
Total score	8⋅4 ± 5⋅38	9⋅8 ± 5⋅90	<0⋅001	8⋅6 ± 5⋅41	9⋅5 ± 5⋅87	<0⋅001	9⋅2 ± 5⋅68	8⋅9 ± 5⋅67	0⋅111	8⋅7 ± 5⋅49	9⋅3 ± 5⋅82	0⋅001
IES-2												
Eating for physical rather than emotional reasons	28⋅4 ± 6⋅45	27⋅9 ± 6⋅46	0⋅012	28⋅5 ± 6⋅56	27⋅9 ± 6⋅36	0⋅005	28⋅0 ± 6⋅59	28⋅4 ± 6⋅29	0⋅050	28⋅2 ± 6⋅55	28⋅1 ± 6⋅38	0⋅613
Unconditional permission to eat	20⋅0 ± 4⋅35	19⋅5 ± 4⋅03	<0⋅001	20⋅0 ± 4⋅45	19⋅5 ± 3⋅97	<0⋅001	19⋅7 ± 4⋅26	19⋅8 ± 4⋅15	0⋅280	19⋅9 ± 4⋅43	19⋅6 ± 4⋅01	0⋅007
Reliance on hunger and satiety cues	21⋅8 ± 5⋅23	21⋅2 ± 5⋅12	0⋅003	21⋅7 ± 5⋅20	21⋅3 ± 5⋅16	0⋅013	21⋅5 ± 5⋅22	21⋅5 ± 5⋅15	0⋅810	21⋅8 ± 5⋅11	21⋅3 ± 5⋅24	0⋅001
Body-food choice congruence	10⋅1 ± 2⋅51	9⋅9 ± 2⋅45	0⋅031	10⋅1 ± 2⋅52	10⋅0 ± 2⋅44	0⋅084	10⋅0 ± 2⋅38	10⋅0 ± 2⋅60	0⋅750	10⋅2 ± 2⋅48	9⋅9 ± 2⋅47	0⋅001
Total score	80⋅4 ± 12⋅21	78⋅7 ± 11⋅41	<0⋅001	80⋅5 ± 12⋅25	78⋅8 ± 11⋅46	<0⋅001	79⋅4 ± 11⋅90	79⋅9 ± 11⋅83	0⋅201	80⋅3 ± 12⋅12	79⋅0 ± 11⋅61	0⋅001

Independent samples *t* test.

EEQ, Emotional Eater Questionnaire; IES-2, Intuitive Eating Scale-2.

The correlation matrix of age, anthropometric measurements with EEQ and IES-2 are shown in Table 5. While there was a weak positive correlation between EEQ and body weight, BMI, waist circumference, waist-height ratio, a weak negative correlation was found between age and waist-hip ratio. There was a weak negative correlation between IES-2 and body weight, BMI, waist-height ratio, waist-hip ratio. In addition, a moderate negative correlation was found between IES-2 and EEQ.

**Table 5.** The correlation matrix of age, anthropometric measurements with EEQ and IES-2

		Age (year)	Body weight (kg)	Waist circumference (cm)	Neck circumference (cm)	BMI (kg/m^2^)	Waist-height ratio	Waist-hip ratio	EEQ	IES-2
Age (year)	*r*	1								
	*P*									
Body weight (kg)	*r*	0⋅329	1							
	*P*	<0⋅001								
Waist circumference (cm)	*r*	0⋅413	0⋅801	1						
	*P*	<0⋅001	<0⋅001							
Neck circumference (cm)	*r*	0⋅322	0⋅671	0⋅685	1					
	*P*	<0⋅001	<0⋅001	<0⋅001						
BMI (kg/m^2^)	*r*	0⋅415	0⋅852	0⋅800	0⋅575	1				
	*P*	<0⋅001	<0⋅001	<0⋅001	<0⋅001					
Waist-height ratio	*r*	0⋅439	0⋅655	0⋅948	0⋅588	0⋅825	1			
	*P*	<0⋅001	<0⋅001	<0⋅001	<0⋅001	<0⋅001				
Waist-hip ratio	*r*	0⋅324	0⋅560	0⋅756	0⋅571	0⋅485	0⋅679	1		
	*P*	<0⋅001	<0⋅001	<0⋅001	<0⋅001	<0⋅001	<0⋅001			
EEQ	*r*	−0⋅095	0⋅086	0⋅047	−0⋅019	0⋅147	0⋅075	−0⋅062	1	
	*P*	<0⋅001	<0⋅001	0⋅004	0⋅244	<0⋅001	<0⋅001	<0⋅001		
IES-2	*r*	−0⋅003	−0⋅064	−0⋅028	−0⋅007	−0⋅116	−0⋅052	0⋅055	−0⋅446	1
	*P*	0⋅426	<0⋅001	0⋅084	0⋅679	<0⋅001	0⋅002	0⋅001	<0⋅001	

Spearman correlation analysis.

BMI, body mass index; EEQ, Emotional Eater Questionnaire; IES-2, Intuitive Eating Scale-2.

## Discussion

In the present study, it was aimed to evaluate the relationship between intuitive eating and emotional eating behaviours in adults with anthropometric measurements of obesity-related disease risk and gender. The main findings of the present study are summarised as follows. The total score and subscales of EEQ were higher in females than males. The scores of the IES-2 subscales and the total score were higher in males than females. In metabolic risk classification according to waist and neck circumference, EEQ scale scores (except type of food) were higher in the metabolic risk group, while IES-2 (except body-food congruence in neck circumference) scores were higher in the non-risk group (*P* < 0⋅05). While there was a positive correlation between EEQ and body weight, BMI, waist circumference, waist-height ratio, a negative correlation was found between age and waist-hip ratio. There was a negative correlation between IES-2 and body weight, BMI, waist-height ratio, waist-hip ratio. In addition, a negative correlation was found between IES-2 and EEQ.

Today, eating behaviours and their effects on health are one of the important issues that are emphasised. The importance of eating behaviours, especially in the prevention of obesity, is known and the eating behaviours of the individuals can be affected by the emotional state, which can affect the individual's eating habits and weight control. The present study aimed to evaluate the relationship between intuitive eating and emotional eating behaviours in adults with anthropometric measurements related obesity.

Although research studies on eating attitudes focused on women, there is no exact information about the difference between emotional and intuitive eating according to gender. It was reported that intuitive eating does not differ according to gender is included in the literature^(16)^. However, a study showed that intuitive eating has been shown to be lower in male participants than in females^(22)^. Also, it was stated that females tend to emotional eating^(13)^ and males tend to eat intuitive eating behaviours^(9,23)^. Similarly, in the present study, while the total and subscales of EEQ scores are higher in female individuals, IES-2 scores are higher in male individuals (Table 1). Emotional eating can be affected by not only gender but also gender, socio-cultural and environmental factors as well as emotional state^(12)^. So these differences may be due to the age, emotional status of the participants in the study.

Obesity is affected by many factors such as age, gender and eating behaviours^(1)^. It is known that with the increase in obesity, anthropometric measurements change and metabolic disease risk increases^(2–4)^. Emotional and intuitive eating behaviours have different effects on obesity and eating attitudes. Emotional eating is associated with an increase in body weight, while intuitive eating is associated with a decrease in body weight^(8,10–12)^. Similarly, in our study, the total EEQ score was higher in obese individuals than in the other BMI groups (Table 2). Although the total score of IES-2 was similar in underweight and normal weight individuals, this score was higher than overweight and obese individuals (Table 2). The score of reliance on hunger and satiety cues were significant both underweight and normal individuals than other groups (*P* < 0⋅001) (Table 2). It has been shown that there is a negative relationship between emotional eating and body weight loss^(14)^. On the other hand, intuitive eating has positive health outcomes including lower BMI and eating disorders^(24)^. And it also focuses on the body's hunger and satiety cues instead of the diet approach^(25)^. Interventions to increase intuitive and decreasing emotional eating behaviours can be effective in preventing both obesity and obesity-related diseases.

These eating behaviours have been shown to be associated with anthropometric measurements related obesity^(9,26)^. However, studies on this subject are limited and conflicted. While there was a weak positive correlation between EEQ and anthropometric measurements (body weight, BMI, waist circumference, waist-height ratio) in this study (Table 5). Singh *et al.*^(27)^ showed a positive relationship between emotional eating and BMI which was a screening tool used to identify obese individuals. However, Özkan *et al.*^(9)^ found a weak negative correlation between IES-2 score and only body weight, they did not find any relationship with BMI, waist circumference and waist-hip ratio. Besides, there were differences in some anthropometric measures related obesity evaluated in emotional eaters and non-emotional eaters in this study. All anthropometric measurements excluding waist-to-hip ratio were higher in male emotional eaters and body weight, BMI, waist circumference and waist-height ratio in female individuals were higher in emotional eaters than non-emotional eaters (Table 3). In addition, a moderate negative correlation was found between IES-2 and EEQ (Table 5). Similarly, it has been reported that emotional eating was inversely associated with intuitive eating^(10)^. There are no clear data on anthropometric measures of both intuitive eating and emotional eating. We think that this study will contribute to the relationship between emotional with intuitive eating behaviour and anthropometric measurements related obesity.

Metabolic risk assessment associated with anthropometric measurements can be an important factor in the prevention of chronic diseases^(5)^. Measures to reduce the existing metabolic risk can help in the fight against chronic diseases^(2,3,5)^. In this study, it is thought that the regulation of eating behaviour may have a positive effect on related anthropometric measurements. While most of EEQ scores are particularly higher in the metabolic risk group, most of IEQ scores are higher in the non-metabolic risk group (*P* < 0⋅05) (Table 4). Only EEQ scale scores (guilt and type of food) were higher in the non-metabolic risk group in risk in accordance with waist-hip ratio (Table 4). Nutritional and psychological treatment to reduce emotional eaters and treatments to increase intuitive eating can contribute to the prevention of metabolic risk and obesity.

## Conclusion

This is the first study evaluated both intuitive eating and emotional eating, and the risk of obesity related to anthropometric measurements to our knowledge. Anthropometric measures have been shown to be associated with emotional eating and intuitive eating. Interventions to increase intuitive and decreasing emotional eating behaviour can be effective in preventing both obesity and obesity-related diseases. Metabolic risk assessment may differ according to eating behaviour. Therefore, a good definition of the eating behaviour of individuals (such as whether they are emotional eater or not) will contribute to reducing metabolic risk. Further research might investigate that randomised controlled studies that will evaluate different eating behaviours with diet intervention groups may be important in the evaluation of eating behaviour and metabolic risk.

### Limitations

There are some limitations in the present study. Firstly, evaluation of body composition as well as anthropometric measurements may be important in the evaluation of eating behaviour. Therefore, it is thought that evaluating body composition in future studies may create a different perspective. Secondly, dietary intake may be investigated in further studies. A study using different anthropometric measurements and calculations other than body weight and BMI, which includes both emotional and intuitive eating behaviour, has not been found in the literature. So, the present study is thought to shed light on the literature.
